# Vegan shrimp alternative made with pink oyster and lion’s mane mushrooms: Nutritional profiles, presence of conjugated phenolic acids, and prototyping

**DOI:** 10.1016/j.crfs.2023.100572

**Published:** 2023-08-19

**Authors:** Flavia Meyer, Aline Hutmacher, Beverly Lu, Nadja Steiger, Laura Nyström, Joan Oñate Narciso

**Affiliations:** a Laboratory of Food Biochemistry, Department of Health Sciences and Technology, ETH Zürich, Schmelzbergstrasse 9, 8092, Zürich, Switzerland; b Department of Food Technology, Engineering and Science, Universitat de Lleida – Agrotecnio CeRCA Center, Avda. Rovira Roure 191, 25198, Lleida, Spain

**Keywords:** Pink oyster mushroom, Lion’s mane, Nutritional profile, Conjugated phenolic acid, Texture analyser

## Abstract

The increasing demand for seafood is responsible for many environmental impacts, especially caused by aquaculture. Shrimp accounts for a substantial part of seafood production and therefore also for negative effects associated with it. This work aimed to develop a mushroom-based shrimp analogue with a texture similar to shrimp using the fruiting bodies of pink oyster mushroom (*Pleurotus djamor*) and lion’s mane (*Hericium erinaceus*). Three flushes of pink oyster mushrooms and a first flush of lion’s mane mushroom were analysed regarding their nutritional composition and whether they are suitable shrimp alternatives. The two mushrooms are rich in proteins (∼32% and ∼26% w/w for the first flush of pink oyster and lion’s mane, respectively). The protein content of pink oyster mushroom decreased and the dietary fibre content increased across the different flushes. The antioxidants in the mushrooms were extracted using different methods, whereby aqueous extracts mostly excelled in terms of antioxidant activity. Hydrolysis confirmed the presence of conjugated *p*-coumaric acid in both mushrooms and possibly conjugated caffeic acid in pink oyster. Texture analysis results of the prototypes were close to the values of fried shrimp. However, although the sensory qualities of the final prototypes were perceived as similar to shrimp, further improvements in the recipe are necessary to make the prototypes indistinguishable from shrimp.

## Highlights

• Lion’s mane and pink oyster mushrooms possess good nutritional quality.
• Conjugated phenolic acids were detected in the mushroom extracts.
• Texture analyses of prototypes closely mimicked shrimp texture.

## Graphical abstract

**Figure:**
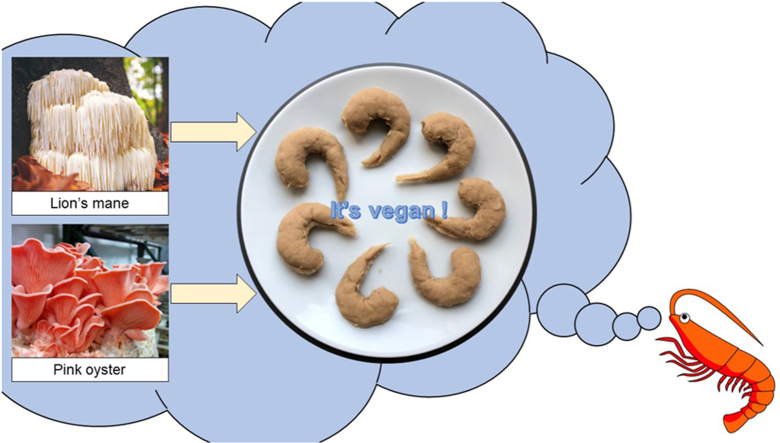

## Teaser

Industrial Relevance: Consumption of seafood like shrimps has massive impacts on the environment as it causes overfishing and bycatch, or water pollution due to aquacultures. To shift consumption to more sustainable choices, comparable alternatives to seafood and shrimps are needed. Shrimps contain high amounts of protein whilst they are low in calories and fat. Mushrooms are suggested as possible substitutions due to their high-quality nutritional profiles. Mushroom-based shrimp analogues with a texture similar to shrimp were then developed. Currently, there is a gap between the consumer demand for more choices of sustainable vegetarian/vegan seafood and plant-based seafood products being sold. The “Mushrimps” will fill this gap and open up a new market of plant-based seafood alternatives that are healthy, nutritious, and sustainable, and which take advantage of locally-grown ingredients.

## 1 Introduction

The worldwide demand for shrimp is constantly increasing, therefore requiring an expansion in seafood production. It is expected that by 2030 the global fish and seafood harvest will reach 187 million tons, with half of the output coming from aquaculture. Shrimp production is projected to increase by 50–60%, accounting for 9% of aquacultural farming (World Bank, 2013). Aquaculture is an efficient and inevitable method to satisfy the growing seafood demand and helps reduce overfishing. Apart from the advantages, intensified aquaculture comes with several adverse side effects. The expansion of shrimp farming requires large areas of land, leading to the deterioration of ecosystems (Radhakrishnan et al., 2021). The wastewater from aquaculture is heavily polluted, posing a threat to humans and the environment. The contamination mainly comes from excessively nutrient-dense feed, fertilizers, antibiotics, as well as excrements and remains from shrimps. A loss in biodiversity and outbreaks of diseases among the shrimps are other potential risks of shrimp farming (Iber and Kasan, 2021). An expansion in seafood production cannot be reached sustainably; therefore, alternatives to fulfil the demand are needed. Animal welfare and a trend towards vegetarian and vegan diets further promote the search for plant-based seafood alternatives. Plant-based meat analogues are already well established, but the development of seafood alternatives is still in the initial phase (Kazir and Livney, 2021). Since seafood belongs to high-cost foods, cheaper imitation products from low-value fish or surimi, which is myofibrillar fish protein, mixed with starch and gelling agents are emerging (Remya et al., 2015). However, these products still consist of fish and do not reduce the negative impacts associated with animal products. Furthermore, these products are neither applicable to a vegetarian or vegan diet, nor can they be consumed in religions renouncing seafood.

Vegetarians and vegans are not the only intended target group for seafood substitute products, but also meat-eaters. To make the substitute products attractive for people who do not want to do without seafood, the analogous should resemble the animal product in texture, taste, and appearance (Kazir and Livney, 2021).

Plant-based analogue products aim to provide a more sustainable alternative to animal products. The search for a suitable plant source for substitute products is essential to improving sustainability. The idea of incorporating mushrooms emerged because the flavour, texture, or colour of certain mushroom species resembles seafood. The pink-orange fruiting bodies of *Pleurotus djamor*, also known as pink oyster mushroom, are reminiscent of the colour of crabs or cooked shrimp. *Pleurotus* species have desirable nutritional values due to their high protein, fiber, and carbohydrate content. Furthermore, they are a good source of vitamins and minerals and are low in calories and fat. The fungi are considered to have several additional benefits, such as antimicrobial, antioxidative, or anti-inflammatory properties (Adebayo and Oloke, 2017). Oyster mushrooms grow predominantly saprophytically in temperate as well as tropical regions. Cultivation is possible on various substrates, including agricultural waste products, straw, or coffee residues (Adebayo and Oloke, 2017; Zadražil, 1978). The tough texture, as well as the flavour of the fungus *Hericium erinaceus*, are reminiscent of crustaceans. The mushroom is commonly known under several names like lion’s mane, hedgehog mushroom or Pom. The terms are derived from the appearance of the mushroom's fruiting body, which is covered in basidiomes in the form of long white spines. *Hericium erinaceus* contains several bioactive compounds and belongs therefore to the medicinal mushrooms. The fungus is part of traditional Chinese medicine, and various pharmaceutical properties are attributed to it. *Hericium erinaceus* grows, like *Pleurotus* species, saprophytically and occurs naturally in Europe, Asia, and North America at temperatures between 18 and 25 °C. The mushroom is often found on dead wood but can be cultivated on artificial substrates and agricultural waste (Thongbai et al., 2015). The pink oyster mushroom and the lion’s mane are not well established in European cuisine. However, they are desirable to be included in the diet due to their nutritional values and potential health benefits. Since these fungi can be cultivated on diverse substrates, nutrients from agricultural waste products can also be used. Moreover, by using waste products as substrate, no edible food is needed for their cultivation. The sustainable cultivation of the mushrooms and their health-promoting compounds make them an appropriate base for meat or seafood analogues.

To impart a texture that is similar to real shrimp, texture-enhancing additives have to be included in the plant-based shrimp analogue. Polysaccharides that are permitted to be applied for thickening, gelling, or further textural improvement of food systems are referred to as “food polysaccharides” (Guimarães et al., 2020). Since these polysaccharides increase the viscosity when dispersed in water, they also belong to the hydrocolloids. Some hydrocolloids, such as starch or xanthan, are applied for thickening. Others, for instance, alginate or carrageenan, can form gels (Saha and Bhattacharya, 2010). The addition of the proper food polysaccharides and hydrocolloids that can form firm gels is important since the inner core of fried shrimp has a gelatinous texture that provides some resistance during biting and chewing.

Our study will test the hypothesis that it is possible to develop mushroom-based shrimp alternatives (“Mushrimps”) using mixtures of mushroom powder from pink oyster mushroom and lion’s mane and food hydrocolloids such as konjac glucomannan and mungbean starch that can mimic the texture of fried shrimp. The aim of our study is to produce a “Mushrimps” prototype that, after cooking, is texturally closer to fried shrimp with a protein content that is similar or close to the protein content of the wet-weight pink oyster and lion’s mane mushrooms.

## 2 Materials and methods

### 2.1 Samples

Mushroom growing kits for pink oyster mushroom and lion’s mane were purchased from Pilzmännchen (Malschwitz, Germany). The pink oyster mushroom and lion’s mane were cultivated on straw and hardwood chips, respectively. Three different flushes of the pink oyster were analysed, each flush representing a different sample. The substrate was not replaced during the analysis. The harvested mushrooms were frozen and freeze-dried in the lyophiliser for 2–3 days. Freeze-dried samples were ground and sieved using a mesh size of 0.355 mm. The lion’s mane from Pilzmännchen was used for detection of conjugated phenolic acids, prototyping, and texture analysis. Additional dried lion’s mane samples were supplied by Mission Mycelium and Stadtpilze. The lion’s mane was cultivated on 50% spent brewers grain consisting of primarily malted barley, and 50% apple wood (Mission Mycelium). The first flush was harvested, dried using circulation air dehydrator at low temperature and ground. The particles were smaller than 0.355 mm. All ground mushroom samples were stored in plastic bags in a desiccator. The samples were analysed in triplicates, except for the dietary fiber analysis, where duplicates were performed.

### 2.2 Proteins

Protein content was analysed by obtaining the total nitrogen content using the standard operating procedure for the TOC-TN analyzer (Shimadzu TOC-LCPH/CPN analyzer, Model TNM-L ROHS). For the extraction of the total nitrogen, 2 mg of sample was accurately weighed into 50-mL tubes. To each tube, 15 mL of Milli-Q water was added and the tubes were vortexed to get rid of any clumps. Afterwards, the tubes were placed in the ultrasonic bath for 10 min. The contents of the tubes were transferred into glass vials with a stirring bar, which were then covered with aluminium foil, and placed into the analyser. A standard NaNO_3_ solution (50 ppm) was measured together with the samples. The parameters were set to 3 out of 5 injections with an injection volume of 40 μL, 5 washes, maximal standard deviation (SD) of 0.1 and maximal coefficient of variation (CV) of 2.00%. To determine the protein content, the TN had to be converted into protein using the conversion factor for mushrooms: 4.4 (Mariotti et al., 2008).

### 2.3 Amino acids

The total proteins were extracted following the protocol of Narciso and Nyström (2020). After the extraction, the tubes were sent to the Functional Genomics Center Zürich for the analysis of the amino acids (Narciso and Nyström, 2020).

### 2.4 β-glucans

The β-glucan content was analysed by commercial kits purchased from Megazyme (Bray, Co. Wicklow, Ireland). The total glucans and α-glucans were measured separately. The β-glucans were then determined by the difference of the total glucans and α-glucans, and the absorbance at 510 nm was measured.

### 2.5 Dietary fibres

The total dietary fibre content was determined using commercial kits purchased from Megazyme (Bray, Co. Wicklow, Ireland). The analyses were performed according to manufacturer’s protocol. Mushroom powder (1 g) was weighed accurately. Two blanks without the mushroom powder were performed simultaneously. Phosphate buffer (50 mL) was added to the samples, and the pH was adjusted to 6.0 ± 0.1. Heat-stable α-amylase (50 μL) was added, and the samples were shaken gently and put in the water bath at 99 °C for 15 min with agitation. The samples were cooled down to room temperature and the pH was increased to 7.5 ± 0.1 using NaOH. Afterwards, protease (100 μL) hydrolysis was initiated. The samples were shaken gently and incubated for 30 min in the water bath at 60 °C with agitation. The pH was adjusted to 4.5 ± 0.2 using HCl and amyloglucosidase (200 μL) was added, and the samples were incubated for another 30 min in the water bath at 60 °C with agitation. The dietary fibres were precipitated using 95% ethanol.

### 2.6 Free soluble sugars

The soluble free sugars were determined using the colourimetric method by Dubois et al. (1956). For the calculation of the soluble sugar mass from the absorbance, the following equation was used (Formula 1):

(1) *m*_*sample*_[***μ****g*] = (*A*_*sample*_−*A*_*blank*_)∗*m*_*standard*_[***μ****g*] (*A*_*standard*_−*A*_*blank*_)

*A*_*standard*_ referred to the absorption average of the five standards at 490 nm and *m*_*standard*_ was equal 50 μg. The result of this equation presented the mass of soluble sugars present in the weighed mushroom sample. To get the mass of the soluble sugars per mass of dry mushroom, the result had to be divided by the exact mass of the mushroom sample that was analysed.

### 2.7 Fats

The fat was extracted with hexane following the procedure of Narciso and Nyström (2020) with some modifications. Mushroom powder (200 mg) was weighed into 50-mL tubes. To each tube, 15 mL of hexane was added using a glass pipette. The tubes were vortexed for 1 min and then put in the ultrasonic bath for 10 min. The samples were centrifuged at 4000 rpm for 10 min. The supernatant was transferred to dried clean glass tubes and then put in the nitrogen evaporator at 60 °C to evaporate the hexane. After the hexane has evaporated, the tubes were cooled down in the desiccator and then weighed again. The difference between this weight and the tared weight of the dry empty tubes represented the amount of fat present in the samples.

### 2.8 Total phenolics

Total phenolic extraction proceeded according to Puttaraju et al. (2006) with slight modifications. For each of the extraction method, 150 mg of mushroom sample were weighed accurately into 50-mL centrifuge tubes. For the water extracts, 10 mL of hot Milli-Q water was added to the sample tubes in a boiling water bath. The methanolic extraction was similar to the water extraction, except that the methanol was not heated prior the extraction. The total phenolic content of the samples was calculated as gallic acid equivalents (GAE).

### 2.9 Conjugated phenolic acids

To test the possibility of the presence of conjugated phenolic acids in the mushrooms, the aqueous extracts (C) from Section 2.8 were further hydrolysed, since it has been reported that mushroom extracts using water as solvent contained the highest amount of phenols (Puttaraju et al., 2006). Moreover, both mushrooms have undergone the same procedure for extraction (C). Each extraction was performed in triplicate.

The hydrolysed extract (H) was obtained from the extract (C) by treating with 2M NaOH, which was then neutralised with 37% HCl solution. To extract the free and released phenolic acids, 5 mL of ethyl acetate was added. After vortexing for a few seconds, the extract was centrifuged at 4000 rpm at 25 °C for 5 min. The upper layer was collected using a glass Pasteur pipette. The ethyl acetate extraction was performed three times in total, whereby the supernatants of the second and third cycle were added to the supernatant of the first one. The hydrolysed extract (H) was stored at 4 °C until use.

The phenolic acids in the mushrooms were further detected using Acquity ultra-performance liquid chromatography (UPLC) coupled to Acquity photodiode array (PDA) detection (254 nm, 280 nm, 325 nm) and Synapt G2 quadrupole time-of-flight mass spectrometry (QTOF-MS) (Waters AG, Baden-Dättwil, Switzerland). The samples were separated using a 2.1 × 100 mm Acquity UPLC BEH C18 column with particle size 1.7 μm (Waters AG, Baden-Dättwil, Switzerland). The injection volume and temperature were set to 2 μL and 26 °C, respectively. Mobile phase consisted of two different solutions: organic eluent A1 (90 : 10: 0.2 – MeOH: Milli-Q water: formic acid) and aqueous eluent B1 (0.2% (v/v) formic acid in water). The flow rate was set to 0.4 mL/min and the elution gradient followed the gradient of the RP-UHPLC analysis, with eluent A1 replacing eluent A and eluent B1 replacing eluent B. Mass spectrometry was operated in negative electrospray ionization (ES) mode and performed for extracts (C) and (H).

### 2.10 Carotenoids

For the extraction of the carotenoids, 100 mg mushroom sample were accurately weighed to dark 50-mL centrifuge tubes and the carotenoid content was measured Narciso and Nyström (2020).

### 2.11 Metals

The minerals were determined by ICP-MS. For this, 250 mg sample were mixed with 4 mL nitric acid (65%) and mineralised by microwave-assisted digestion. The mineralised samples were diluted to 50 g solution using Milli-Q water. For potassium and phosphorous determination, the samples were further diluted with a dilution factor of 50. The concentrations of the minerals were measured by ICP-MS (iCap RQ) in helium (He) collision mode. A blank and certified reference material (Wheat Flour NIST 1567b) were processed and analysed along the samples.

### 2.12 Texture analyser

Textural analysis was performed with a texture analyser (TA.XT plus Texture Analyser, Stable Micro System, UK). A Texture Profile Analysis (TPA) was conducted to determine hardness and chewiness. The stress, defined as force per area, was calculated using the hardness derived from the TPA and the contact area of the sample. The cutting force was tested as a second texture analysis test to determine the peak positive force and the high strain modulus. However, the cutting force testing was only implemented at the end of the texture finding process.

For the TPA test, the probe used was the 100 mm compression plate, and the 5 kg load cell was inserted. In the T.A. settings library, TPA was selected under the special tests. The pre-test speed was set to 1 mm/s, and the test speed as well as the post-test speed to 5 mm/s “Strain” was selected as the target mode and set to 30%. The time was set to 5 s. The trigger type was left at automatic (force) and the trigger force at 0.049 N. The advanced options were turned off, and the simplified TPA macro was run during the testing. The sample shape was defined as cylindrical and the measured sample diameter and height, as well as the calculated stress area, were inserted.

The cutting force was measured with the fake-warner-bratzler blade probe and the 50 kg load cell. In the T.A. settings, "return to start” was selected, and compression as test mode was defined. The pre-test and test speeds were set to 1 mm/s and the post-test speed to 10 mm/s. The target mode was set to distance, and the distance was adjusted for every prototype individually, depending on its height. The distance was always set to a value of 3–5 mm more than the height of the prototype to ensure that the probe cuts through the entire sample. The trigger type was left at automatic (force), and the trigger force was set to 5 g. The advanced options were turned off. The Young’s modulus initial and height strain macro was run during the testing.

### 2.13 Prototype

The final prototype has the following composition: 12% mungbean starch, 10% mushroom powder, 8% konjac glucomannan, and 2% sugar, in water. The mushroom powder was dissolved in water and heated. After cooling to room temperature, the remaining ingredients (konjac glucomannan and sugar) were added, and the mixture was transferred into moulds. The formed prototypes were steamed over boiling water for 15 min and either microwaved at the highest level and fried or directly fried for 5 min at medium heat. The time in the microwave varied between 20 and 35 s, and the texture was analysed after microwaving as well as after the additional step of frying. The TPA test was performed with cylindrical pieces cut out of the prototypes with an average diameter of 18 mm and a high of 10 mm.

### 2.14 Statistical analysis

ANOVA was used for statistical analysis of differences within a mushroom species, followed by Tukey’s HSD or Tukey-Kramer for pairwise comparison. T-test was used for statistical analysis of differences between the aqueous and hydrolysed extracts. Significance was given at p < 0.05 level.

## 3 Results and discussion

### 3.1 Protein and essential amino acids

The total nitrogen of the samples was determined and converted into protein content using the conversion factor 4.4. The total protein content and the essential amino acid profiles are shown in Table 1.

**Table 1:** Protein content (%w/w) and essential amino acid (a.a.) profile of all mushroom samples.

	Pink Oyster flush 1	Pink Oyster flush 2	Pink Oyster flush 3	Lion’s Mane
Protein content	31.51 ± 2.79^b^	15.76 ± 0.84^a^	15.34 ± 1.27^a^	26.03 ± 3.01^b^
Essential a.a. (%)				
Histidine^a^	2.13 ± 0.12^a^	2.02 ± 0.03^a^	2.1 ± 0.05^a^	2.26 ± 0.06^a^
Threonine^a^	5.79 ± 0.15^a^	5.82 ± 0.10^a^	5.87 ± 0.23^a^	7.22 ± 2.16^a^
Lysine^a^	6.34 ± 0.25^a^	6.45 ± 0.08^a^	6.37 ± 0.13^a^	6.33 ± 0.15^a^
Tyrosine^a^	2.42 ± 0.12^a^	2.44 ± 0.13^a^	2.53 ± 0.07^a^	2.45 ± 0.01^a^
Methionine	1.27 ± 0.01^b^	1.06 ± 0.06^a^	1.25 ± 0.10^b^	1.03 ± 0.02^a^
Valine	6.86 ± 0.05^a^	7.22 ± 0.02^b^	7.07 ± 0.07^ab^	7.02 ± 0.18^ab^
Isoleucine	4.7 ± 0.02^a^	5.16 ± 0.02^c^	4.87 ± 0.02^b^	4.56 ± 0.12^a^
Leucine	8.03 ± 0.04^a^	8.42 ± 0.09^b^	8.19 ± 0.04^ab^	8.28 ± 0.23^ab^
Phenylalanine	3.48 ± 0.21^a^	4.23 ± 0.02^c^	3.88 ± 0.03^b^	3.66 ± 0.06^ab^

Data is expressed as Mean ± SD (n = 3). Different superscript letters represent significance at p < 0.05 using Tukey’s HSD.

a indicates no significant difference across the samples at p < 0.05.

As for many nutrients, the protein content varies amongst different mushroom species (Ogundana and Fagade, 1982). In the case of lion’s mane and the first flush of the pink oyster mushroom, the protein content was equal to 26.03 ± 3.01% and 31.51 ± 2.79%, respectively (Table 1). Similar contents were reported from previous studies investigating the same mushrooms (Raman et al., 2021; Cheung, 2013). Compared to other mushroom species, lion’s mane and pink oyster mushroom had higher measured protein content, but still within the expected range of 20–30% (Naeem et al., 2020). It is noticeable that for the later flushes, the protein content decreased by half, resulting in 15.76 ± 0.84% for the second flush and 15.34 ± 1.27% for the third flush. These differences from flush 1 to later flushes were significant. A possible reason for this decrease could be that the majority of the nitrogen present in the substrate was used to generate the fruiting bodies of the first flush. Therefore, there was less nitrogen left for later flushes. This is consistent with observations that nitrogen-rich substrates might enhance the protein content of mushrooms (Naraian et al., 2009).

Except for tryptophan and cysteine, all essential amino acids were detected. Since essential amino acids cannot be synthesised by the human body, their presence contributes to high-quality protein. The most abundant amino acids in all the mushroom samples were aspartic (10.90–11.28%) and glutamic acid (10.51–11.28%), followed by glycine (8.80–9.55%), alanine (8.79–9.18%), and leucine (8.03–8.42%). Free amino acids present in shrimps have been shown to function as a major contributor to seafood flavour. Investigations on the taste of shrimps have shown that higher umami, sweetness, and overall flavour were exhibited in sea water shrimps. These shrimps were characterised by high concentrations of glycine (32.71%), arginine (23.96%), alanine (9.59%), and proline (7.31%) (Liang et al., 2008). The concentrations of alanine and proline found in lion’s mane and pink oyster mushroom are comparable to the ones found in shrimp. Additionally, the mushrooms are also high in glycine and alanine, which could contribute to a suitable flavour as shrimp alternative (Liang et al., 2008).

Due to their amino acid profile and high protein content, lion’s mane and pink oyster mushrooms show big potential regarding their use as functional food ingredients and meat or fish alternatives. First flushes of the mushrooms contained highest amounts of protein. Therefore, if mushrooms were to be used as food ingredients and as alternative protein sources, it is preferable to use the first flushes.

### 3.2 Dietary fibres, β-glucans and free soluble sugars

The amount of β-glucans, dietary fibres, soluble sugars are presented (Table 2).

**Table 2:** Content of beta-glucan, dietary fibres and soluble sugars (in %w/w) from all mushroom samples.

Sample	β-Glucan [%w/w]	Dietary Fibre [%w/w]	Soluble Sugar [%w/w]
Pink Oyster flush 1	19.37 ± 0.96^a^	29.64 ± 3.44^a^	0.09 ± 0.01^b^
Pink Oyster flush 2	29.71 ± 1.12^c^	46.68 ± 3.17^b^	0.06 ± 0.02^ab^
Pink Oyster flush 3	33.35 ± 1.04^d^	46.67 ± 3.30^b^	0.06 ± 0.04^ab^
Lion’s Mane	22.1 ± 1.11^b^	39.23 ± 0.55^ab^	0.03 ± 0.00^a^

Data is expressed as Mean ± SD (n = 2 for all dietary fibre; n = 3 for β-glucans and soluble sugars). Different superscript letters represent significance at p < 0.05 using Tukey’s HSD.

In all carbohydrate fractions, the soluble sugar constituted the smallest component of carbohydrates with less than 1% w/w for all mushroom samples (Table 2). The soluble sugar content decreased with the number of flushes, although these values were not significantly different. However, there was an observable significant difference between pink oyster and lion’s mane species. Whilst the soluble sugar content in the first flush of pink oyster mushroom was 0.09%, the lion’s mane contained only 0.03% of soluble sugars. Pink oyster mushrooms contained higher levels of soluble sugars than lion’s mane mushrooms. Sugar content between different mushroom species can vary. Reported sugar content found in other oyster mushroom species ranged from ∼5.0% of fresh weight to 64.9% of fresh weight (Agarwal et al., 2018; Reis et al., 2012). Compared to these amounts, the obtained results for the lion’s mane and pink oyster mushrooms were extremely low.

As for most nutrients, the content of β-glucans varies amongst different species. The β-glucan amount of 19.37 ± 0.96% w/w was determined for the first flush of the pink oyster mushroom. This is significantly less than the obtained β-glucan content of 22.10 ± 1.11% w/w in lion’s mane (Table 2). Hence, lion’s mane can provide more health benefits related to β-glucans. The results for β-glucan content in both mushrooms are comparable to what is suggested in literature (Cerletti et al., 2021; Chaiyasut et al., 2018). The amount of β-glucans in the pink oyster mushrooms increased with the number of flushes. In the case of the first flush, β-glucans contributed 19.37 ± 0.96% to the dry matter, whilst in the second and third flushes, β-glucans accounted for 29.71 ± 1.12% and 33.35 ± 1.04%, respectively. The substrate gets older with increasing number of flushes. Hence, the growth conditions of the mushroom changed, which can result in a change in the nutritional composition. A possible explanation for the shift to higher amounts of β-glucans in later flushes could be that more β-glucans were synthesised. The biosynthesis of β-glucans is an enzymatic process catalysed by the β-(1,3)-glucan synthetase (Papaspyridi et al., 2018). The affinity of glucan synthetase to its substrate, and therefore the activity of the enzyme, depends on the fungal species, their developmental stage, and the environmental conditions (Ruiz-Herrera, 1991). As the substrate of the mushroom gets older, it contains less nutrients than in the previous flushes. This shortage in nutrients would display a stress factor for the mycelia, which can lead to an activation of stress response mechanisms. One possible response could be the generation of thicker cell walls, which correlates with higher amounts of β-glucans, since they are constituents of mushroom cell walls (Ruiz-Herrera, 1991). Another possible explanation for the increase in β-glucan content could be the stimulation of the β-(1,3)-glucan synthetase. The activity of this enzyme can be stimulated or inhibited by certain molecules such as ATP and GTP, or divalent metals (Papaspyridi et al., 2018). It is possible that the mycelia accumulated such molecules and metals across the different flushes. This could have led to an increase in β-glucan synthesis.

High dietary fibre levels were detected in lion’s mane and pink oyster mushrooms. The dietary fibre content varied amongst the different flushes of the pink oyster mushroom, as well as between the mushroom species. There was a significant difference in the dietary fibre content between the first flush of pink oyster mushroom and the second and third flushes, and the lion’s mane mushroom. The first flush contained 29.64 ± 3.44%, clearly the lowest amount of dietary fibre amongst all the mushroom samples tested. The second and third flushes contained the highest amounts of dietary fibre with 46.68 ± 3.17% and 46.67 ± 3.30%, respectively, although there was no significant difference between these two values. The increase in the amount of dietary fibre from the first flush to the later flushes is similar to the increasing trend in the amounts of β-glucans. This is possible since β-glucans are part of the dietary fibre and changes in β-glucan content can affect the amount of total dietary fibre. Results obtained in this study are comparable to previous investigations of the dietary fibre content in mushrooms. In the case of the lion’s mane, a dietary fibre content of 34.0 ± 1.0% was reported (Cheung, 2013), whilst for the pink oyster mushroom, a value of 37.0 ± 2.6% was obtained (Nile and Park, 2014). Due to the many health benefits of dietary fibres and β-glucans and their high amounts in lion’s mane and pink oyster mushrooms, there is potential for applying these mushrooms as functional food ingredients.

The fact that the contents of dietary fibre and proteins changed inversely across the different flushes must be considered from the perspective of using mushrooms as shrimp alternatives. Whilst high protein content is important, high amounts of dietary fibre and β-glucan have beneficial health properties. Therefore, there is a trade-off between these two nutrients. A possible solution could be the mixture of different mushrooms powders. This combines the properties of the different flushes, resulting in a high protein content as well as good amounts of beneficial dietary fibres. It must be considered that later flushes generated lower yields than the first flush, which is why from an economical point of view, first flushes are preferred.

### 3.3 Fats

Fat content in pink oyster mushroom ranged from 1.48 ± 0.04% for flush 1 to 1.68 ± 0.11% in flush 3 (Table 3). For the lion’s mane, fat content was 3.12 ± 0.48%.

**Table 3:** Fat content (%w/w) of mushroom samples.

Samples	Fat (%w/w)
Pink Oyster flush 1	1.48 ± 0.04^a^
Pink Oyster flush 2	1.53 ± 0.10^a^
Pink Oyster flush 3	1.68 ± 0.11^a^
Lion’s Mane	3.12 ± 0.48^b^

Data is expressed as Mean ± SD (n = 3). Different superscript letters represent significance at p < 0.05 using Tukey’s HSD.

There is an insignificant increase in fat content in the later flushes of pink oyster mushroom. The present results are consistent with previous studies where 1.72% fat content was reported for pink oyster mushroom (Raman et al., 2020) and 2.3–3.5% fat content for the lion’s mane (Friedman, 2015). There was a significant difference between the species, as the lion’s mane contained approximately twice the amount of fat than the pink oyster mushroom. In the case of shrimp, the fat content is low, ranging from 0.8 to 1.3% (wet weight) (Li et al., 2021; Sriket et al., 2007). Therefore, the idea of generating an alternative to shrimps made from mushrooms is nutritionally beneficial in terms of fat content.

### 3.4 Total phenolics

The total phenolic contents of the lion’s mane mushroom and three different flushes of the pink oyster mushroom were investigated as GAE in mg/g of dried mushroom (Table 4).

**Table 4:** GAE [mg/g] of water and methanol extracts from all mushroom samples.

	GAE [mg/g]	
Sample	Water extracts	Methanolic extracts
Pink Oyster flush 1	13.05 ± 0.46^b^	8.02 ± 0.35^b^
Pink Oyster flush 2	12.83 ± 0.11^b^	9.39 ± 0.06^c^
Pink Oyster flush 3	11.79 ± 0.19^a^	7.41 ± 0.08^a^
Lion’s Mane	11.62 ± 0.16^a^	9.43 ± 0.04^c^

Data is expressed as Mean ± SD (n = 3). Different superscript letters represent significance at p < 0.05 using Tukey’s HSD.

The water extracts ranged from 11.62 to 13.05 mg/g of dry weight, whilst the methanolic extracts ranged from 7.41 to 9.43 mg/g of dry weight (Table 4). For all mushrooms, the total phenolic content resulted in higher amounts when the extraction was done with water. The difference between the water extracts and the methanolic extracts were significant for all mushroom samples.

Water is a more polar solvent than methanol. Since more phenolics were detected in water extracts, it can be suggested that the majority of the phenolic compounds extracted from the mushroom samples were hydrophilic/polar. This is consistent with results shown in other literature, where in the case of the pink oyster mushroom, the difference was even higher (13.3 mg/g for water extracts and 3.6 mg/g for methanolic extracts) (Puttaraju et al., 2006). A previous study investigating subfractions of methanolic extracts from lion’s mane mushrooms showed that more phenolic compounds were extracted when using more polar subfractions of methanolic extracts than when using less polar subfractions (Li et al., 2012). In the case of the lion’s mane, the difference between the water and methanolic extracts was less than that of the pink oyster mushroom. The amount of phenolic compounds in the methanolic extracts of the lion’s mane was high. This suggests that the lion’s mane contained relatively more non-polar compounds than the pink oyster mushroom.

The polarity of phenolic compounds is related to the antioxidant activity. The difference in polarity depends on the hydroxyl groups attached to the aromatic ring (Galanakis et al., 2013). Higher number of hydroxyl groups leads to an increase in polarity and therefore in the antioxidant activity (Milić et al., 1998). This would suggest higher antioxidant activity of pink oyster mushrooms. Based on the obtained results, it seems that the amount of polar phenolics, and therefore the antioxidant activity, decreased with the number of flushes.

### 3.5 Conjugated phenolic acids

**Fig. 1:**
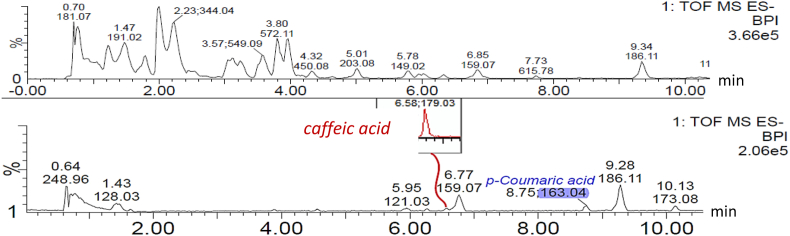
UPLC-MS spectrum of pink oyster replicate (C) (top) and pink oyster hydrolysed replicate (H) (bottom) containing conjugated *p*-coumaric acid and possibly conjugated caffeic acid. (For interpretation of the references to colour in this figure legend, the reader is referred to the Web version of this article.)

The phenolic acid *p*-coumaric acid with m/z = 163.040 and retention time of around 8.8 min at 325 nm was found in all hydrolysed replicates of extract (H). Caffeic acid with m/z = 179.035 and retention time of around 6.6 min at 325 nm was detected in the hydrolysed pink oyster replicate (H) (Fig. 1).

The presence of conjugated phenolic acids in plants has been confirmed by several researchers (Ahmad et al., 2016; Gao et al., 2017). However, studies on the phenolic acids in fungi is mostly limited to free phenolic acids. Hydrolysis of the aqueous extracts (C) confirmed the presence of conjugated phenolic acids in pink oyster and lion’s mane. More precisely, conjugated *p*-coumaric acid was found in both mushroom species and traces of possibly caffeic acid were detected in pink oyster. These findings suggest that the majority of phenolic acids are conjugated. In fact, the detected amounts in extracts (H) greatly exceeds the amount of free (Çayan et al., 2020) and conjugated *p*-coumaric acid (Mattila et al., 2001; Yildiz et al., 2015) in several mushroom species. The concentration of conjugated *p*-coumaric acid in pink oyster and lion’s mane is also comparable to the amount of conjugated *p*-coumaric acid in barley. However, compared to potato and berries, the mushrooms exhibit lower levels (Pei et al., 2016).

### 3.6 Carotenoids

The samples were analysed for their lutein, zeaxanthin, β-cryptoxanthin, and β-carotene content. Amongst the investigated carotenoids, β-carotene was the only one that was not detected in any of the samples. The amounts of total carotenoids ranged from 12.28 ± 0.2 μg/g for of the second flush of the pink oyster mushroom to 19.25 ± 2.13 μg/g in lion’s mane (Table 5).

**Table 5:** Concentrations of single and total carotenoids as μg/g of dry matter.

Samples	Lutein	Zeaxanthin	β-cryptoxanthin	β-carotene	Total carotenoids
Pink Oyster flush 1	4.29 ± 0.06^a^	4.85 ± 0.10^ab^	6.60 ± 0.37^ab^	–	15.74 ± 0.22^ab^
Pink Oyster flush 2	4.69 ± 1.12^a^	3.31 ± 0.72^a^	4.28 ± 0.20^a^	–	12.28 ± 0.20^a^
Pink Oyster flush 3	4.50 ± 0.29^a^	4.81 ± 0.47^ab^	6.97 ± 0.34^b^	–	16.28 ± 1.03^ab^
Lion’s Mane	7.22 ± 0.73^b^	6.23 ± 0.93^b^	5.80 ± 1.24^ab^	–	19.25 ± 2.13^b^

Data is expressed as Mean ± SD (n = 3; except for pink oyster flush 1 and 2 with n = 2). Different superscript letters represent significance at p < 0.05 using Tukey’s HSD.

Because carotenoids are not widespread in mushroom (Kalač, 2013), high contents were not expected. The second flush of the pink oyster mushroom contained the lowest amounts of total carotenoids, zeaxanthin, and β-cryptoxanthin; however, these values were only significantly different from those of the third flush, not from those of the first flush. Due to its colour, it could have been expected that pink oyster mushroom contains higher amounts of carotenoids than the lion’s mane. Comparing the results, this does not seem to be the case, since the lion’s mane contained the highest amounts of lutein and total carotenoids. Previous studies reported amounts of 47.81 μg/g and 45.98 μg/g of β-carotene in methanolic and ethanolic extracts, respectively, for lion’s mane mushroom (Ghosh et al., 2021). This is not consistent with the present results, where β-carotene was not detected. This discrepancy could be due to the different extraction and detection methods. The mentioned study used methanol and ethanol for extraction and performed spectrophotometry as detection method. The generally low amounts of carotenoids found in all the mushroom samples suggests that the antioxidant activity of the mushrooms can be attributed more to phenolic compounds.

### 3.7 Metals

Many important minerals such as Na, Mg, Ca, and Fe were detected in the mushroom samples. Most abundant minerals found in all mushroom samples were K (2.7 × 10^4^ to 3.5 × 10^4^ μg/g of dry matter) and P (1.0 × 10^4^ to 1.4 × 10^4^ μg/g of dry matter) (Table 6). Magnesium (Mg) was also present (1.1 × 10^3^ to 1.3 × 10^3^ μg/g). The least abundant minerals detected were heavy metals such as (6.8 × 10^−3^ to 22.6 × 10^−3^ μg/g of dry matter) and Pb (0.1–0.4 μg/g).

**Table 6:** Concentrations of minerals [μg/g of dry matter] in mushroom samples.

	Pink Oyster flush 1	Pink Oyster flush 2	Pink Oyster flush 3	Lion’s Mane
Na	10.9 ± 0.8	23.7 ± 5.2	22.8 ± 0.8	187.8 ± 4.0
Mg	1.1 × 10^3^± 0.0	1.3 × 10^3^± 0.0	1.3 × 10^3^± 0.0	1.2 × 10^3^± 0.0
Al	0.9 ± 0.2	1.8 ± 0.2	1.4 ± 0.3	1.1 ± 0.0
P	1.4 × 10^4^± 0.0	1.1 × 10^4^± 0.0	1.0 × 10^4^± 0.0	1.3 × 10^4^± 0.0
K	3.5 × 10^4^± 0.0	2.7 × 10^4^± 0.0	2.7 × 10^4^± 0.0	2.7 × 10^4^± 0.0
Ca	59.1 ± 1.0	239.6 ± 1.3	326.2 ± 2.2	70.2 ± 2.0
Mn	15.6 ± 0.2	13.6 ± 0.1	13.5 ± 0.0	27.7 ± 0.1
Fe	164.2 ± 0.6	98.4 ± 1.3	120.6 ± 8.6	111.7 ± 1.3
Cu	17.4 ± 0.2	13.2 ± 0.2	11.0 ± 0.2	25.3 ± 0.2
Zn	176.9 ± 2.3	110.9 ± 2.4	102.2 ± 1.6	124.6 ± 1.6
As	6.8 × 10^−3^ ± 2.0 × 10^−3^	17.1 × 10^−3^ ± 2.5 × 10^−3^	22.6 × 10^−3^ ± 0.7 × 10^−3^	19.3 × 10^−3^ ± 0.9 × 10^−3^
Se	0.2 ± 0.0	0.2 ± 0.0	0.2 ± 0.1	0.1 ± 0.0
Cd	1.1 ± 0.0	0.8 ± 0.0	0.8 ± 0.0	0.5 ± 0.0
Pb	0.0 ± 0.0	0.1 ± 0.1	0.1 ± 0.0	0.4 ± 0.2

Data is expressed as Mean ± SD (n = 3).

There were differences between lion’s mane and pink oyster mushrooms for most minerals. Remarkable differences can be observed for Na with 10.9 ± 0.8 μg/g vs 187.8 ± 4.0 μg/g, As with 6.8 × 10^−3^ ± 2.0 × 10^−3^ μg/g vs 19.3 × 10^−3^ ± 0.9 × 10^−3^ μg/g, and Pb with 0.0 ± 0.0 μg/g vs 0.4 ± 0.2 μg/g for the first flush of the pink oyster and the lion’s mane mushroom, respectively. Due to their toxicity and health risks (Wang et al., 2005), presence or high concentrations of Pb and As is not desirable in food. Tolerable daily intakes for Pb and As are 3.57 μg/kg body weight and 0.42 μg/kg body weight, respectively (Mohamed et al., 2017). For both these heavy metals, the lion’s mane mushroom contained higher amounts compared to the pink oyster mushroom. However, the amounts for these heavy metals in both mushroom species were very low. For example, an adult weighing 60 kg would have to consume over 500 g dry mass of lion’s mane mushroom to exceed the tolerable daily intake for Pb. This would correspond to a consumption of over 3 kg of lion’s mane mushroom per day. In the case of As, the needed amount to exceed the tolerable intake would be even higher. Such high intakes are not considered as realistic and therefore, the amounts of As and Pb present in the mushroom should not pose any risks. For some minerals, there seemed to be a decrease over the several flushes of the pink oyster mushroom. For example, the amount of P decreased from 1.4 × 10^4^ ± 0.0 μg/g in the first flush to 1.1 × 10^4^ ± 0.0 μg/g in the second flush, and to 1.0 × 10^4^ ± 0.0 μg/g in the third flush. Phosphorus is a key nutrient for the growth of the mushroom, but most of the phosphorous in soils and substrates is unavailable for uptake due to its fixation as insoluble calcium phosphate (Malhotra et al., 2018). A possible explanation for the decrease in phosphorous content of later flushes could be that the accessible P was consumed by earlier flushes.

It has already been discussed that the amount of β-glucan seems to increase with the number of flushes and that the stimulation of the β-(1,3)-glucan synthetase could be a possible reason. It is reported that Mg^2+^, Mn^2+^, Ca^2+^, and Fe^2+^ are some of the divalent metals that are able to stimulate the β-(1,3)-glucan synthetase (Papaspyridi et al., 2018). The efficiency of the stimulation ability of different divalent metals depends on the mushroom species. Based on the results of the β-glucan and Ca quantification, it could be suggested that the β-(1,3)-glucan synthetase of the pink oyster mushroom could have been stimulated by the high Ca^2+^ content.

Both mushroom species contained many essential minerals. Compared to green tiger shrimps, lower levels of Ca and Na were present in the mushrooms, but the mushrooms contained higher amounts of K, P, and Fe (Yanar and Çelik, 2006). Since Ca and P are important for the formation of bones and teeth (Soetan et al., 2016), it is crucial that sufficient amounts of these minerals are consumed. Green tiger shrimps contain approximately ten times higher amounts of Ca than the investigated mushrooms. If the mushrooms were to be used as alternatives to shrimps, this needs to be considered. Overall, both mushroom species contained good amounts of minerals that are essential for physiological functions, except for Ca, which is still significantly higher in shrimps.

### 3.8 Prototype

Using pink oyster and lion’s mane mushroom powder and plant-based polysaccharides, prototypes of the shrimp alternative were developed. The final prototype was treated with various heating methods to receive a texture similar to real shrimp. The peak positive force and the high strain modulus were higher for prototypes with microwave treatment in addition to steaming compared to only steaming (Table 7). The highest values were received with prototypes that were steamed, heated up in the microwave for 35 s and afterwards fried for 5 min. The high strain modulus was not significantly different between the fried prototypes that were 35 s in the microwave and the prototypes with a 20 s microwave treatment. However, the peak positive force showed a significant deviation between these prototypes.

**Table 7:** Results derived from cutting force test performed on the final prototype.

Prototype treatment	Peak positive force [N]	High strain modulus [Pa]
**Raw**	5.04 ± 0.14^c^	20.32 ± 3.86^c^
**Steamed**	8.74 ± 0.17^c^	33.96 ± 2.48^c^
**steamed + fried**	18.81 ± 1.31^b^	68.36 ± 3.70^b^
**steamed + microwave (35 s)**	20.86 ± 1.31^b^	86.51 ± 3.93^b^
**steamed + microwave (35 s) + fried**	32.58 ± 5.29^a^	122.89 ± 4.71^a^
**steamed + microwave (20 s) + fried**	21.34 ± 1.93^b^	120.03 ± 25.90^a^

Data are presented as Mean ± SD (n = 3). Different superscript letters represent significance at p < 0.05 using Tukey’s HSD.

**Fig. 2:**
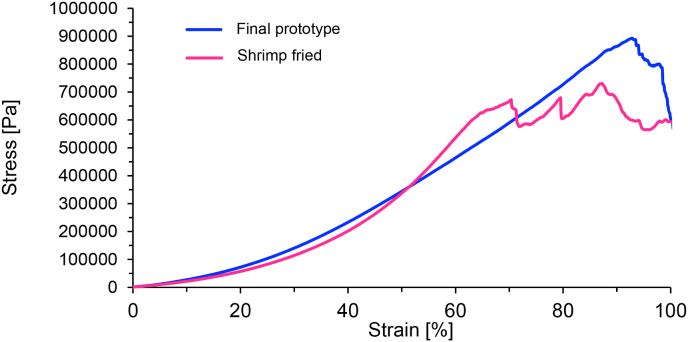
Stress-strain curve from the cutting force testing of final prototype (steamed + microwaved 35 s + fried) and fried shrimp. Data are presented as the mean of three replicates, respectively.

The stress-strain curve for the steamed, 35 s microwaved, and fried prototype showed a similar slope to the curve of fried shrimp (Fig. 2). However, the curve of the prototype was rather a straight line, whereas the graph of the fried shrimp was more curved. The stress applied to the fried shrimp was lower in the beginning than the stress applied to the prototype for the same strain. However, from 50% strain exceeded the stress-strain curve of the prototype the curve of the fried shrimp.

The hardness as well as the stress, determined with the TPA test, decreased with increasing heat treatment (Table 8). Prototypes that were microwaved after steaming had lower hardness and stress values than prototypes that were fried after steaming. The chewiness was lower for prototypes fried after steaming compared to those without frying. Prototypes with microwave treatment after steaming showed higher chewiness than the only steamed prototypes. The additional step of frying after steaming and microwave treatment led to an increase in chewiness.

**Table 8:** Hardness, chewiness, and stress derived from TPA test of the final prototype.

Prototype treatment	Hardness [N]	Chewiness	Stress [MPa]
**Steamed**	25.92 ± 1.45^a^	2241.59 ± 209.91^a^	114.03 ± 6.40^a^
**steamed + fried**	19.49 ± 1.65^b^	1725.59 ± 253.49^a^	76.60 ± 6.49^b^
**steamed + microwave**	12.37 ± 0.98^c^	2462.30 ± 759.61^a^	63.93 ± 5.05^bc^
**steamed + microwave + fried**	10.58 ± 0.72^c^	2657.55 ± 33.95^a^	46.60 ± 3.16^c^

Data are presented as Mean ± SD (n = 3). Different superscript letters represent significance at p < 0.05 using Tukey’s HSD.

The strain modulus of the final prototype was lower than the modulus of shrimp. Modulus is defined as the slope of the stress-strain curve in the linear region and calculated therefore through the division of stress by strain. The modulus is a parameter for the elasticity of the sample, which describes the tendency to deform upon compression. A higher value for the modulus of elasticity indicates a stiffer material (Askeland, 1996). The maximum force needed for cutting through the steamed, microwaved, and fried prototype was comparable to the force required for fried shrimp. However, the stress-strain curve showed a steeper slope for the shrimp than the prototype, which indicates a less elastic texture of the shrimp.

Prototypes where the mushroom-water slurry was heated had a firmer texture than prototypes where this step was omitted. The disparity in texture between prototypes with and without the additional heating step might come from the thermal denaturation of mushroom proteins. Denatured proteins can increase the viscosity and, therefore, probably enhance the firmness of the prototype (Liu et al., 2004).

The steaming of the prototypes initiated the gelatinisation of the starch, which resulted in a firm texture upon cooling (Belitz et al., 2008). Konjac glucomannan and other hydrocolloids increase the viscosity of starch gels and decrease the time until the maximum viscosity is reached. The increase in density might be due to interactions between starch and hydrocolloids or their competition for water (Bahnassey and Breene, 1994). However, no synergistic interactions between konjac glucomannan and starch occurred. The konjac glucomannan did not form a gel since it requires an alkaline coagulant, such as sodium carbonate, for this purpose (Zhou et al., 2014). It is presumed that konjac glucomannan initially promotes retrogradation but prevents syneresis during storage (Yoshimuraa et al., 1998). The enhanced retrogradation upon cooling probably contributed to the firm texture of the prototypes.

Microwaving generates a high heat causing the outward flow of water. The water on the surface evaporated upon withdrawal of the prototype from the microwave and cooling at room temperature, thus decreasing the moisture content and increasing the firmness (Chandrasekaran et al., 2013). The cutting force results for the steamed, microwaved, and fried prototype with a 35 s microwave time were higher compared to the prototype with a 20 s microwave time. The prolonged microwaving potentially caused a greater loss in moisture, hence raising the firmness of the prototype. Frying further increased the peak positive force and high strain modulus, resulting in an elasticity similar to fried shrimp.

In contrast to the cutting force results, the hardness of the prototype and accordingly, the stress, decreased with heat treatment. The cylindrical prototypes were treated equally to the shrimp-shaped prototypes, though the difference in shape and size might have caused dissimilar textural changes upon the heating steps. Additionally, the prototypes were covered with cling wrap while still being warm and tested in warm conditions. Only the steamed prototypes were left to cool down entirely before the texture analysis. Reheating the prototypes might have induced gelatinisation of retrograded starch and remaining starch that has not been gelatinised in the first heating step, reducing the viscosity directly after the reheating (Han et al., 2009). The cling wrap further contributed to keeping the prototypes warm, slowing down the retrogradation and reducing the evaporation of water. This might explain the reduced hardness with additional heating steps.

## 4 Conclusion

Lion’s mane and pink oyster mushrooms were found to possess good nutritional quality due to their high protein content, amino acid profile, total dietary fibre including β-glucan content, total phenolic and mineral content and amount of fat. Although pink oyster and lion’s mane may not provide the tested mushroom-based products with extensive antioxidant properties, the nutritional composition of mushrooms does correspond to a healthy diet. The results support the presumption of mushrooms as a plant-based and healthy alternative to shrimp. Texture analysis results of the prototypes were close to the values of the shrimp evaluation. However, sensorially the final prototypes were perceived as similar but still clearly different compared to shrimp. Therefore, further textural improvements to the prototypes are required. Furthermore, taste and appearance must be improved. The development of a shrimp alternative holds great importance as resource exploitation must be kept at a minimum to ensure food security.

## Funding

This research was generously and kindly supported by the NTN Innovation Booster *Swiss Food Ecosystems* for the project “Mushrimps”. The funding source had no involvement in the study design, in the collection, analysis and interpretation of data, in the writing of the report, and in the decision to submit the article for publication.

## CRediT authorship contribution statement

**Flavia Meyer:** Conceptualization, Data curation, Formal analysis, Investigation, Validation, Visualization, Writing – original draft. **Aline Hutmacher:** Conceptualization, Data curation, Formal analysis, Investigation, Validation, Visualization, Writing – original draft. **Beverly Lu:** Conceptualization, Data curation, Formal analysis, Investigation, Validation, Visualization, Writing – original draft. **Nadja Steiger:** Conceptualization, Data curation, Formal analysis, Funding acquisition, Investigation, Methodology, Supervision, Validation, Writing – review & editing. **Laura Nyström:** Project administration, Resources, Writing – review & editing. **Joan Oñate Narciso:** Conceptualization, Data curation, Formal analysis, Funding acquisition, Investigation, Methodology, Supervision, Validation, Writing – review & editing.

## Declaration of competing interest

None.

## Data Availability

Data will be made available on request.
